# Contemporary Theory and Research

**Published:** 1893-06-10

**Authors:** 


### Contemporary Theory and Research.

### UNHEALTHY EMPLOYMENTS.

We learn from the new *Labour Gazette* that the Home Secretary has thought it desirable to institute special inquiries into certain occupations and manufactures where there is good reason for thinking that the processes are dangerous to the health of the workpeople. These inquiries relate to (1) the various processes connected with lead, and white lead in chief, (2) potteries, (3) chemical works, and (4) quarries. They will be conducted by certain of H.M. inspectors, assisted by medical and other experts. Their primary object is to assist the Home Secretary in the framing of special rules under section 8 of the Factory Act of 1891, but the reference to each committee is wide enough to admit of a thorough investigation into the various processes concerned. The names of the gentlemen who compose the committee are added, and are a sufficient guarantee that the investigations will be carried through with all the thoroughness and scientific knowledge demanded by the large interests affected.

### CARBONATE OF GUAIACOL.

Carbonate of guaïacol is a fine crystallised powder without smell or taste, insoluble in water. The point of fusion is at 90 deg. In contact with soda it decomposes when cold into its constituent parts—carbonic acid and guaïacol. This preparation is advocated by M. Seifert and M. Hoelscher as a remedy for tuberculosis. They have made trial of carbonate of guaïacol in the treatment of sixty tuberculosis patients, and prefer it to creosote for many reasons. It is inoffensive to the digestive organs, and has no effect upon a healthy stomach. But where in the tuberculosis patient a great quantity of saprophyte bacteria are present in the intestines, the carbonate of guaïacol decomposes more rapidly, giving off free guaïacol, which hinders the development of the bacteria, and finally destroys them altogether. The above doctors maintain that in the blood of persons affected with tuberculosis there exist, beside the normal albuminoids, others of a pathological nature. It is these which occasion the fever, night sweats, &c., they unite with the guaïacol in the blood, and form thus inoffensive substances. Thanks to this process the fever disappears, as well as the night sweats, anorexia, dyspepsia, &c. Being without smell or taste, the preparation is taken without disgust or resistance by the most fastidious patient; it is also free from all irritating effect on the mucous membrane.

### OLD AGE PENSIONS IN FRANCE.

The question of providing for the aged poor and chronic invalids has been recently before the Municipal Council of Paris. The inadequacy of the provision supplied by the Assistance Publique is shown by a report declaring that 1,500 vacancies only were available among 4,500 applicants. A strong expression of opinion was manifested against the unnecessary removal of old people from their families to institutions, and it is urged that a choice should be given wherever possible of receiving an annual pension. By this system of "out-door relief" it is hoped that the "hospices" already over full may be reserved for old people without families, for paralytics, and those whose maladies require such constant attention as to constitute a serious burden on their friends. The extension of the out-patient departments of the hospitals and "maisons de secours," in order to provide for the needs of the chronically ill and infirm, has also its advocates.

